# Serum exosomal microRNAs as predictive markers for EGFR mutations in non–small‐cell lung cancer

**DOI:** 10.1002/jcla.23743

**Published:** 2021-03-08

**Authors:** Junbo Xia, Man Luo, Lujun Dai, Liusheng Wang, Limin Wang, Jing Zhu

**Affiliations:** ^1^ Department of Pulmonary Medicine Affiliated Hangzhou First people’s Hospital Zhejiang University School of Medicine Hangzhou China; ^2^ Department of Infectious Diseases Affiliated Hangzhou First people’s Hospital Zhejiang University School of Medicine Hangzhou China

**Keywords:** EGFR, exosome, miRNAs, mutant, NSCLC, RNA sequencing, wild type

## Abstract

**Background:**

Current therapeutic drugs show positive effects on non–small‐cell lung cancer (NSCLC) patients with mutant epidermal growth factor receptor (EGFR) expression, whereas a lesser beneficial effect is generally noted on NSCLC patients with wild‐type EGFR. Therefore, identification of new detection methods for the accurate clinical diagnosis of NSCLC is essential.

**Methods:**

In this study, tumor‐derived exosomes from the plasma of EGFR mutation and wild‐type NSCLC patients were isolated. Extensive exosomal miRNA profiling of EGFR mutation and wild‐type NSCLC patients, in comparison with healthy individuals, was performed using miRNA‐sequencing analysis.

**Results:**

The variation of exosomal miRNA expression between control group (NR) and NCSLC samples (AM and AW) was identified. 96 significantly different expressed miRNAs were identified. Of these, 39 miRNAs were upregulated and 57 were downregulated. 11 miRNAs were downregulated, and 31 miRNAs were upregulated in the miRNA expression between NR and AM. Compared with healthy donors, 54 upregulated miRNAs and 36 downregulated miRNAs were observed in samples from AW patients. 40 different expressed miRNAs were identified in AM samples, compared with AW. Ten of upregulated miRNAs are miR‐260, miR‐1169, miR‐117, miR‐15b‐5p, miRNA‐731, miR‐342‐5p, miR‐ 898, miR‐1384, miR‐56, and miR‐1214. Ten of downregulated miRNAs are miR‐99b‐5p, miR‐1116, miR‐689, miR‐818, miR‐604, miR‐72, miR‐955, miR‐403, miR‐1228, and miR‐836.

**Conclusion:**

The exosomal miR‐1169 and miR‐260 as potential candidates, which contain specific characteristics that can distinguish between wild‐type EGFR and mutant EGFR NSCLC patients in early‐stage cancers.

## Graphical abstract

In this study, we isolated tumor‐derived exosomes from the plasma of EGFR mutation and wild‐type NSCLC patients. Extensive exosomal miRNA profiling of EGFR mutation and wild‐type NSCLC patients, in comparison with healthy individuals, was performed using miRNA‐sequencing analysis. Exosomal miR‐1169 and miR‐260 were identified as potential candidates, which contain specific characteristics that can distinguish between wild‐type EGFR and mutant EGFR NSCLC patients in early‐stage cancers.

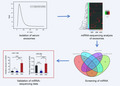

## 1 INTRODUCTION

Lung cancer remains the leading cause of cancer deaths in China^1^ and United States.^2^ Non–small‐cell lung cancer (NSCLC) is the most common type of lung cancer, accounting for about 85% of global cases. Late diagnosis and delayed treatments are the major obstacles to improving lung cancer outcomes, with an average 5‐year survival rate of approximately 15% for NSCLC.^1^, ^3^ In NSCLC patients, 93% of mutations in the gene encoding epidermal growth factor receptor (EGFR) occurs in exons 18–21 and more than 80% in Del19 and L858R. The remainder are rare mutations, including G719X, E709X, Del18, and Ins19 in exon 18, Ins20 and S768I in exon 20, and L861Q in exon 21.^4^ In view of the EGFR mutations in NSCLC, the development of EGFR tyrosine kinase inhibitors (EGFR‐TKIs), such as gefitinib, afatinib, and oxetinib, has demonstrated enhanced therapeutic effects in NSCLC patients with EGFR mutations. In contrast, EGFR‐TKIs have shown poor therapeutic effects in NSCLC patients with wild‐type EGFR.^5^, ^6^ Therefore, while early diagnosis is important, NSCLC differentiation is essential in the early management of NSCLC patients to identify the appropriate treatments strategies. Tissue biopsy represents the gold standard for the diagnosis of lung cancer and for genotyping mutant EGFR.^7^, ^8^ However, due to the invasive nature of the cancer and metastatic state, some tissue samples are difficult to obtain, as well as non‐surgical resection tissues not being representative of the whole tumor. Therefore, tissue biopsies may not fulfill the requirements of early screening, dynamic follow‐up, accurate, and comprehensive responses to the biological properties of the lung cancer.

MicroRNAs (miRNAs) are reported to play important roles in gene regulation and are detected in many organisms.^9^ Accumulating studies demonstrate the involvement of miRNAs in tumorigenesis and development processes, such as cancer cell metastasis, hematopoiesis, proliferation, and cell death. In addition, miRNAs have been extensively studied in the diagnosis and prognosis of lung cancer. Expression of miR‐137, miR‐372, let‐7, miR‐221, and miR‐182 in lung cancer may be used as markers for predicting the survival rate of lung cancer patients.^10^, ^11^ Microarray data showed that 32 miRNAs are highly expressed in adenocarcinoma tissues. Moreover, miRNAs let‐7e, miR‐25, miR‐191, miR‐34a, and miR‐34c are associated with prognosis.^12^ The role of circulating miRNAs in non‐invasive diagnosis has been extensively studied^13^, ^14^; however, few studies have identified the specific miRNA profiles of mutant EGFR and wild‐type EGFR in early NSCLC diagnosis using peripheral blood.

With the advancement of more accurate detection methods in medicine, liquid biopsy has shown great promise due to the availability of samples for analysis. The three main targets of liquid biopsy include, circulating tumor DNA (ctDNA), circulating tumor cells (CTC), and exosomes. Among these, exosomes were discovered in 1983 and more recently have been the focus of extensive research within the liquid biopsy industry due to its wide distribution, high content, and stable structure. Exosomes are composed of 30–100 nm extracellular vesicles. Nucleotides and proteins secreted by specific cell types found in various body fluids, participated in communication between cells.^15^ Unlike circulating miRNAs, exosomes are enriched within the circulatory system and prevent RNase degradation.^16^ Identification of exogenous miRNA in body fluids confirms their potential as biomarkers for the clinical diagnosis or prognosis of NSCLC.^17^, ^18^ Twelve exosomal miRNAs are reported to be specifically expressed in adenocarcinoma patients with lung cancer compared with the control group, indicating a close correlation between circulating miRNA expression of tumor‐derived exosomes and tumor miRNA.^19^ Moreover, the expression of exosomal let‐7f, miR‐20b, miR‐301, miR‐379, and miR‐200b in plasma or bronchoalveolar lavage is reported to differentiate NSCLC patients from healthy individuals.^20^ Based on these published observations, we sought to further determine whether exosomal miRNAs could differentiate between NSCLC patients with wild‐type and mutant EGFR. Therefore, the objective of this study was to identify tumor‐derived exosomal biomarkers which are able to discriminate between EGFR mutation and wild‐type NSCLC patients during the early stages of diagnosis, in order to improve the specificity and sensitivity of NSCLC diagnosis using this non‐invasive method.

In this study, tumor‐derived exosomes from the plasma of EGFR mutation and wild‐type NSCLC patients were isolated. An extensive exosomal miRNA profiling of EGFR mutation and wild‐type NSCLC patients paired with healthy individuals was performed using miRNA‐sequencing.

## 2 MATERIALS AND METHODS

### 2.1 Clinical sample collection

Serum samples of NSCLC patients in stages II‐IV were collected from the the Hangzhou First People's Hospital, Zhejiang University School of Medicine. The study was approved by ethics committees of the hospital and was conducted in accordance with the Declaration of Helsinki and the International Conference on Harmonization Good Clinical Practice guidelines. All patients provided written informed consent before participating in the study. Of the 64 patients enrolled for testing and validations studies, 32 had EGFR wild‐type NSCLC and 32 had EGFR mutant NSCLC, between stages II‐IV, respectively. Twenty healthy individuals matched for sex and age in the testing and validation sets were included in the study. A symptomatic set of patients were included, who were suspected of suffering with NSCLC according to preliminary diagnosis, in order to verify the diagnostic accuracy of the selected miRNA panels. Detailed clinical data are summarized in Table 2. Plasma samples of patients were collected in vacuum blood tubes with anticoagulant prior to surgery or pharmacotherapy and handled within 1 h after collection. All individuals gave written consent for the use of their plasma samples and pathologic information to be used in this research. Patients in this study had no other malignant disorders, and the serum samples were nonhemolytic, obtained by centrifuging 2 ml peripheral blood at 280 g for 10 min. Supernatants were stored at – 80°C until further analysis.

### 2.2 Cell lines and culture

BEAS2B, HCC827, NCI‐H1299, NCI‐H1650, and A549 cell lines were obtained from the American Type Culture Collection. Cells were cultured in high‐glucose Dulbecco's Modified Eagle's Medium (DMEM, Hyclone), supplemented with 10% fetal bovine serum (Gibco) and penicillin‐streptomycin (Beyotime) at 37°C with 5% CO_2_.

### 2.3 Transmission electron microscopy

Exosomes samples were diluted to 0.5 mg/ml with phosphate‐buffered saline (PBS), and spotted onto a glow‐discharged copper grids covered with filter paper, and left to dry for 10 min under an infrared lamp. Finally, exosomes were stained with a drop of 1% phosphotungstic acid (Sigma, USA) for 1 min and dried using the infrared lamp. Exosomes were observed under transmission electron microscopy (TEM; JEM‐1010 microscope) at 80 kV.

### 2.4 Isolation of exosomes and extraction of RNA

Ultracentrifuge methods were utilized. The supernatant was centrifuged further at 110,000 *g* for 70 min. All steps were performed at 4°C. The pellets were resuspended in PBS for TEM. For patient serum sample, exosomes were extracted from 500 μl serum using a Total Exosome Isolation Kit (Invitrogen) following the manufacturers’ protocol. Isolated exosomes were fully suspended in 100 μl PBS and then mixed with 1 ml of RNAiso reagent (Takara). Finally, the RNAs were dissolved in 20 μl RNase‐free water. The quality of RNA was determined using a NanoDrop 2000 (Thermo) by OD260/280 and stored at −80°C until further analysis.

### 2.5 Quantitative RT‐PCR

Complementary DNA (cDNA) of miRNA was synthesized using the PrimeScript RT Reagent Kit (TaKaRa). Stem‐loop quantitative reverse transcriptase polymerase chain reaction (qRT‐PCR) assays using TaqMan miRNA probes (Thermo) were subsequently conducted to quantitate the levels of mature miRNAs. For serum exosomes, microRNA‐16 was used as an internal control in the qRT‐PCR analysis. qRT‐PCR reactions were performed on the real‐time PCR system in 96‐well plates at 95°C for 10 min, followed by 40 cycles at 95°C for 15 s, 60°C for 1 min and finally added a melting curve analysis at 95°C for 15 s, 60°C for 1 min, and 95°C for 15 s. Each sample was analyzed in duplicate.

### 2.6 Western blot

The proteins of exosomes were extracted with RIPA buffer (Beyotime, China) and centrifuged at 11,809 g for 15 min at 4°C. A small amount of supernatant was quantified using the bicinchoninic acid assay (BCA) kit (Beyotime), and the remaining proteins were separated by SDS‐PAGE and transferred onto a PVDF membrane (Millipore) according to standard methods. After blocking with 5% non‐fat milk for 2 h, the membrane was incubated overnight at 4°C with primary antibodies for CD63 (proteintech). After washing the non‐bound primary antibodies, the membrane was incubated with secondary HRP‐conjugated anti‐mouse or anti‐rabbit antibodies (Beyotime). The antigen‐antibody reaction was detected by a chemiluminescence HRP substrate kit (Millipore).

### 2.7 Small RNA sequencing libraries preparation

Small RNA sequencing was performed using Illumina HiSeq (Illumina). Total RNA of each sample was extracted using TRIzol Reagent (Invitrogen) and miRNeasy Mini Kit (Qiagen). Total RNA of each sample was quantified and qualified by Agilent 2100 Bioanalyzer (Agilent Technologies), NanoDrop (Thermo) and 1% agrose gel. Total RNA, 2 μg, with a RIN value above 7.5 was used for subsequent library preparations. Next‐generation sequencing library preparations were constructed according to the manufacturer's protocols (NEBNext® Multiplex Small RNA library Prep Set for Illumina®). The 3 ´SR Adaptor for Illumina was ligated to the small RNA using 3´ Ligation Enzyme. To prevent adaptor‐dimer formation, the excess of 3 ´SR Adaptor was hybrid with SR RT Primer for Illumina. 5 ´SR Adaptor for Illumina was ligated to the small RNA using 5´ Ligation Enzyme and the first strand of cDNA was synthesized using ProtoScript II Reverse Transcriptase. Each sample was then amplified by PCR for 12 cycles using P5 and P7 primers, with both primers carrying sequences which can anneal with flow cell to perform bridge PCR and P7 primers carrying a six‐base index, to allow multiplexing. The PCR products of ~140 bp were recovered and cleaned up using PAGE, validated using an Agilent 2100 Bioanalyzer (Agilent Technologies), and quantified by Qubit 2.0 Fluorometer (Invitrogen). Libraries with different indexes were multiplexed and loaded on the Illumina HiSeq instrument according to manufacturer's instructions. Sequencing was carried out using a 1 × 50 single‐end (SE)/2 × 150 paired‐end (PE) configuration; image analysis and base calling were conducted by the HiSeq Control Software (HCS) and OLB and GAPipeline‐1.6 (Illumina) on the HiSeq instrument. The sequences were processed and analyzed by GENEWIZ.

### 2.8 Data analysis

Quality control of samples was performed to remove technical sequences, including adapters, PCR primers, or fragments thereof, quality of bases lower than 20 was passed through a FASTQ filter and the data were processed using the Cut adapt software (version 1.9.1)to ensure clean high quality data.

The miRDeep2 software was used to identify novel miRNAs from deep sequencing, and obtaining all the identified microRNA expression data.

For differential expression analysis, the DESeq/DESeq2 Bioconductor package was used, based on the negative binomial distribution. After adjustment using Benjamini and Hochberg's approach for controlling the false discovery rate, the *p* value <0.05 was set to detect differential expressed miRNAs.

Gene Ontology (GO)‐TermFinder was used to identify GO terms, annotating a list of enriched genes with *p* value <0.05 defined as significant.

KEGG (Kyoto Encyclopedia of Genes and Genomes) is a collection of databases dealing with genomes, biological pathways, diseases, drugs, and chemical substances (http://en.wikipedia.org/wiki/KEGG). In‐house scripts were used to enrich significant differential expression gene in KEGG pathways.

For microRNA target mRNA analysis, miRanda detection software was used to predict the microRNA‐mRNA in animals.

### 2.9 Statistical analysis

Results are expressed as the mean  ± standard error of the mean (SEM). Student's *t* test, the Mann‐Whitney *U* test and chi‐square test were used to compare the differences among different groups. Receiver operator characteristic (ROC) curve and the area under curve (AUC) were performed to estimate the diagnostic accuracy. A *p* < 0.05 value was considered statistically significant. All statistical analyses were carried out using SPSS 22.0 and GraphPad Prism 8.

## 3 RESULTS

### 3.1 Identification of exosomes and determination of miRNA in serum

**FIGURE 1:**
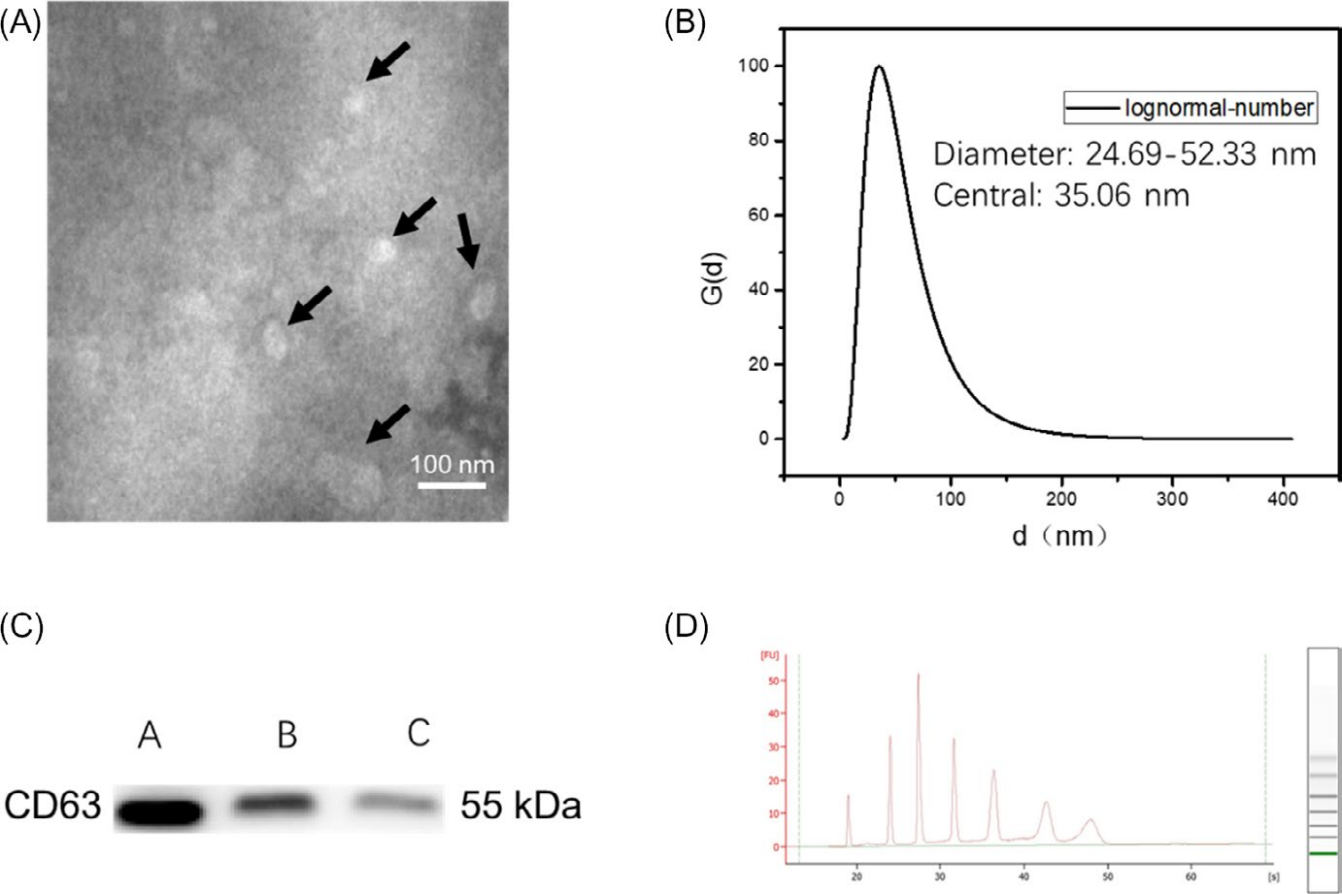
Identification of exosomal microRNAs isolated from serum samples. (A) Exosomes purified from serum were observed under TEM (Scale bar: 100 nm). (B) Nanoparticle tracking analysis of 50× diluted serum exosomes by DLS. (C) Exosomes were purified in serum samples from patients with NSCLC using the ExoQuick system and subsequently lysed. Lanes 1–3: Different protein concentrations (1.0, 0.5, and 0.2 μg/ml) of purified exosome lysates were separated by polyacrylamide gel electrophoresis, transferred to membranes, and blotted with anti‐CD63 immunoglobulin G. (D) The quality of purified exosomes using the ExoQuick system was determined using an Agilent Bioanalyzer (Agilent Technologies)

To determine the efficacy of the methods used to isolate the serum exosomes, the exosomal marker was analyzed by Western blot and exosomal morphology of microvesicles was characterized by TEM. Serum samples from NSCLC patients with wild‐type or mutant EGFR were obtained before treatment. Exosomes were obtained using the ExoQuick exosome purification kit and observed under TEM. TEM analysis revealed that the microvesicles separated from the serum were a round or elliptical membranes (Figure 1A). Vesicle size ranged between 30 and 100 nm and displayed a uniform appearance (Figure 1B). CD63, a tetraspanin family member which localizes internally in exosomal vesicles, were expressed in isolated serum exosomal pellets as specific bands, but not in the exosomes with depleted serum supernatant (Figure 1C). These results demonstrated that the separated serum particles exhibit exosome characteristics, confirming the successful exosome separation techniques employed here.

A variety of RNAs, such as messenger RNAs (mRNAs), ribosomal RNAs (rRNAs), transfer RNAs (tRNAs), and miRNAs, were identified in exosomes. Therefore, it is essential to establish strategies for the accurate detection of miRNAs in exosomes. Here, miRNAs were purified from isolated serum exosomes using miRNA isolation kits. From this, RNAs were subsequently isolated based on size, revealing twenty‐five nucleotide‐long small RNA representing the miRNAs. Contrarily, 18S and 28S ribosomal RNA were barely detectable (Figure 1D). These results confirmed the specificity of our experimental methods for obtaining miRNAs and further suggest that miRNAs isolated from serum exosomes may be suitable and valuable for clinical sample analysis.

### 3.2 miRNA‐sequencing analysis of exosomes

**FIGURE 2:**
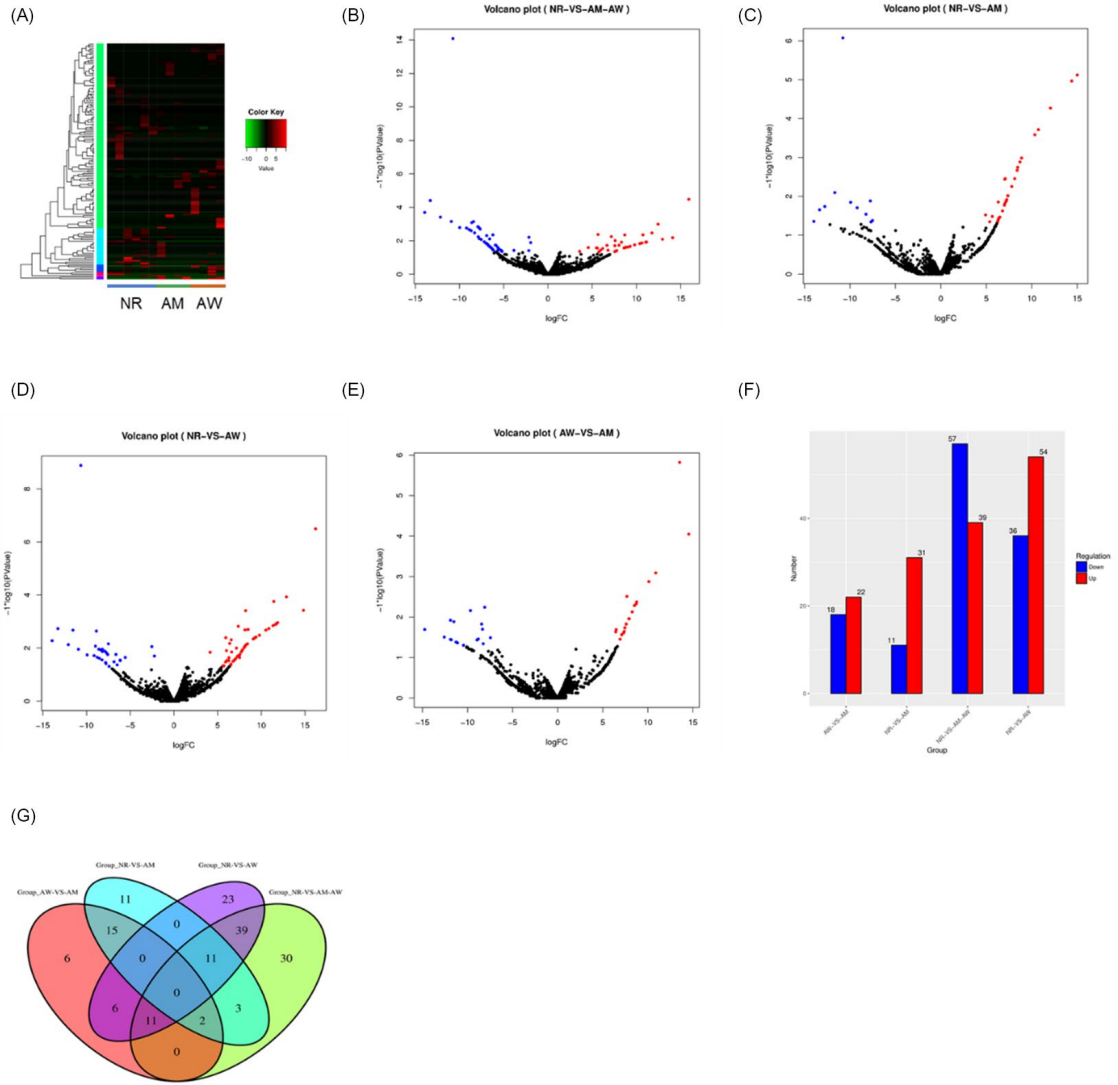
Identification of EGFR wild‐type NSCLC (AW)‐ and EGFR mutant NSCLC (AM)‐specific miRNAs from miRNA‐sequencing data. (A) Heat map of pairwise correlation values of samples in the test set. Pearson correlation coefficients were calculated for log2 transformed ratios of the miRNAs panels with more than 1000 RPM in healthy individuals and in AW and AM patients. (B–E) Volcano plot of normalized miRNA expression levels for miRNAs showing statistically differential expressions between healthy control, AW, and AM samples. (F) Histogram. (G) The Venn diagram identifies overlapping and non‐overlapping miRNAs in healthy controls, AM and AW samples, that are upregulated or downregulated in comparison with healthy subjects in the testing group. miRNAs with a twofold change, either positive or negative, were included

Of the 8 patients with NSCLC, 4 presented with wild‐type EGFR samples (AW) and 4 were mutant EGFR subjects (19 depleted or L858R in 21 exon, AM). Six healthy volunteer samples (NR) were used as the control group. Among the 8 NSCLC patients, 6 did not undergo treatment whereas 2 patients had received chemotherapy. The median age in the NSCLC group (69.5 ± 8.6 years) was older than that of the control groups (57.5 ± 15.3 years); however, no significant differences in gender were noted among both groups. The primary content of total exosomes from NSCLC patients was obtained from the tumors within the plasma. Subsequent qRT‐PCR validation on the total extracted exosomes from NSCLC patients with wild‐type or mutant EGFR, as well as healthy donors was performed (Figure S1A and B). Exosomal miRNA sequence analysis performed with next‐generation sequencing techniques revealed 2284 miRNAs. The thermogram, histogram, venn, and volcano plots showed the variation of exosomal miRNA expression between control group (NR) and NCSLC samples (AM and AW) (Figure 2A–G). In total, 96 significantly different (*p* < 0.05) expressed miRNAs with greater than twofold changes were identified. Of these, 39 miRNAs were upregulated and 57 were downregulated (Figure 2A, B and F). Only 11 miRNAs were downregulated and 31 miRNAs were upregulated in the volcano plots and histogram, showing variation in the miRNA expression between NR and AM (Figure 2A, C and F). Compared with healthy donors, 54 upregulated miRNAs and 36 downregulated miRNAs were observed in samples from AW patients (Figure 2A, D and F). Interestingly, 40 different expressed miRNAs were identified, including 18 downregulated miRNAs and 22 upregulated miRNAs in AM samples, compared with AW (Figure 2A, E, and F). These identified miRNAs include miR‐142‐5p, miR‐592, miR‐217, miR‐451B, and miR‐150, which have also been previously reported as NSCLC diagnostic or prognostic biomarkers. Moreover, miR‐142‐5p and miR‐592 are expressed in both AW and AM NSCLC–derived exosomes.

Further analysis revealed 11 markedly different miRNA expression in both the NR compared with the AW group, and the NR compared with the AM group (Figure 2G). Among these miRNAs, seven upregulated miRNAs and four downregulated miRNAs in both groups were observed (Figure 2F, Table 1). Ten of these exosomal miRNAs showed the same trend in NR compared with AW, and NR compared with AM groups, with regard to expressions of miR‐142‐5p, miR‐106b‐3p, miR‐504, miR‐996, miR‐3158‐3p, miR‐940, miR‐548ad‐5p, miR‐422, miR‐1273, and miR‐715 (Table 1). Thus, these serum exosomal miRNAs may potentially serve to act as biomarkers for NSCLC; however, may not be suitable in distinguishing between AW and AM. Expression of miR‐592 in AW samples was 10–20 times higher than that detected in AM samples (Table 1). Therefore, the serum exosomal level of miR‐592 can potentially identify AW within NSCLC patients. Moreover, we observed specific upregulated expression of miR‐1169 in AW NSCLC patients (Table 1) compared with AM. In healthy donors, no significant differences were observed in NSCLC patients with AM. Decreased expression of miR‐260 was also noted in AW whereas no significant change was observed in AM patients (Table 1). Therefore, the serum exosomal level of miR‐1169 and miR‐260 can be used as biomarkers to differentiate between AW and AM in NSCLC patients.

**TABLE 1:** miRNA profiling between AM‐ and AW‐dependent exosomal miRNAs

miRNA regulation (*p* < 0.05)			
AW vs. healthy controls		AM vs. healthy controls	
hsa‐miR‐142‐5p	hsa‐miR‐1‐3p	hsa‐miR‐142‐5p	NovelmiRNA‐226
NovelmiRNA‐689	hsa‐miR‐1‐5p	NovelmiRNA‐1169	NovelmiRNA‐489
NovelmiRNA‐592	NovelmiRNA‐948	hsa‐miR‐106b‐3p	NovelmiRNA‐1015
NovelmiRNA‐72	NovelmiRNA‐1143	NovelmiRNA‐117	hsa‐miR‐5100
NovelmiRNA‐604	NovelmiRNA‐470	NovelmiRNA‐592	NovelmiRNA‐1058
hsa‐miR‐106b‐3p	NovelmiRNA‐1123	NovelmiRNA‐996	NovelmiRNA‐70
NovelmiRNA‐1116	hsa‐miR‐324‐3p	NovelmiRNA‐504	NovelmiRNA‐422
NovelmiRNA‐955	NovelmiRNA‐199	hsa‐miR‐3158‐3p	NovelmiRNA‐1078
NovelmiRNA‐403	NovelmiRNA‐706	NovelmiRNA‐1384	NovelmiRNA‐818
NovelmiRNA‐836	NovelmiRNA‐731	NovelmiRNA‐56	NovelmiRNA‐1273
NovelmiRNA‐1228	NovelmiRNA‐561	NovelmiRNA‐71	NovelmiRNA‐715
NovelmiRNA‐217	NovelmiRNA‐358	NovelmiRNA‐1219	
NovelmiRNA‐40	NovelmiRNA‐334	NovelmiRNA‐216	
NovelmiRNA‐90	NovelmiRNA‐1064	NovelmiRNA‐838	
NovelmiRNA‐395	NovelmiRNA‐650	NovelmiRNA‐150	
NovelmiRNA‐254	NovelmiRNA‐539	hsa‐miR‐548ad‐5p	
NovelmiRNA‐504	NovelmiRNA‐21	NovelmiRNA‐67	
NovelmiRNA‐617	NovelmiRNA‐9	hsa‐miR‐29b‐3p	
NovelmiRNA‐602	NovelmiRNA‐814	NovelmiRNA‐940	
hsa‐miR‐451b	NovelmiRNA‐1214	NovelmiRNA‐1303	
NovelmiRNA‐597	NovelmiRNA‐1344	hsa‐miR‐1273f	
NovelmiRNA‐86	hsa‐miR‐342‐5p	NovelmiRNA‐691	
NovelmiRNA‐1006	NovelmiRNA‐167	NovelmiRNA‐886	
NovelmiRNA‐670	NovelmiRNA‐748	NovelmiRNA‐357	
NovelmiRNA‐996	NovelmiRNA‐402	NovelmiRNA‐146	
NovelmiRNA‐116	NovelmiRNA‐898	NovelmiRNA‐151	
NovelmiRNA‐464	hsa‐miR‐15b‐5p	NovelmiRNA‐1168	
hsa‐miR‐3158‐3p	NovelmiRNA‐832	NovelmiRNA‐523	
NovelmiRNA‐1280	NovelmiRNA‐1125	NovelmiRNA‐298	
NovelmiRNA‐623	NovelmiRNA‐492	NovelmiRNA‐1218	
NovelmiRNA‐450	NovelmiRNA‐422	hsa‐miR‐548ac	
NovelmiRNA‐940	NovelmiRNA‐129		
NovelmiRNA‐649	NovelmiRNA‐260		
NovelmiRNA‐1124	NovelmiRNA‐1310		
NovelmiRNA‐54	NovelmiRNA‐1273		
NovelmiRNA‐59	NovelmiRNA‐715		
NovelmiRNA‐964			
hsa‐miR‐548ad‐5p			
NovelmiRNA‐1271			
NovelmiRNA‐667			
NovelmiRNA‐1170			
NovelmiRNA‐1028			
NovelmiRNA‐554			
NovelmiRNA‐761			
hsa‐miR‐6747‐5p			
NovelmiRNA‐583			
NovelmiRNA‐998			
NovelmiRNA‐887			
NovelmiRNA‐429			
NovelmiRNA‐1082			
NovelmiRNA‐240			
NovelmiRNA‐1017			
NovelmiRNA‐94			
hsa‐miR‐4440			

### 3.3 Validation of miRNA‐sequencing data by quantitative RT‐PCR analysis

Circulating miRNAs are studied extensively as cancer diagnostic biomarkers in lung cancer and other types of carcinomas. However, target expressions are inconsistent in serum, plasma, or other bodily fluids.^21^, ^22^, ^23^ Previous studies have demonstrated the enrichment of miRNAs in exosomes.^24^, ^25^, ^26^, ^27^ In this study, the expression of selected miRNAs, miR‐1169, and miR‐260 was analyzed in tumor‐derived exosomal and circulating plasma, due to their important roles in tumorigenesis.

**FIGURE 3:**
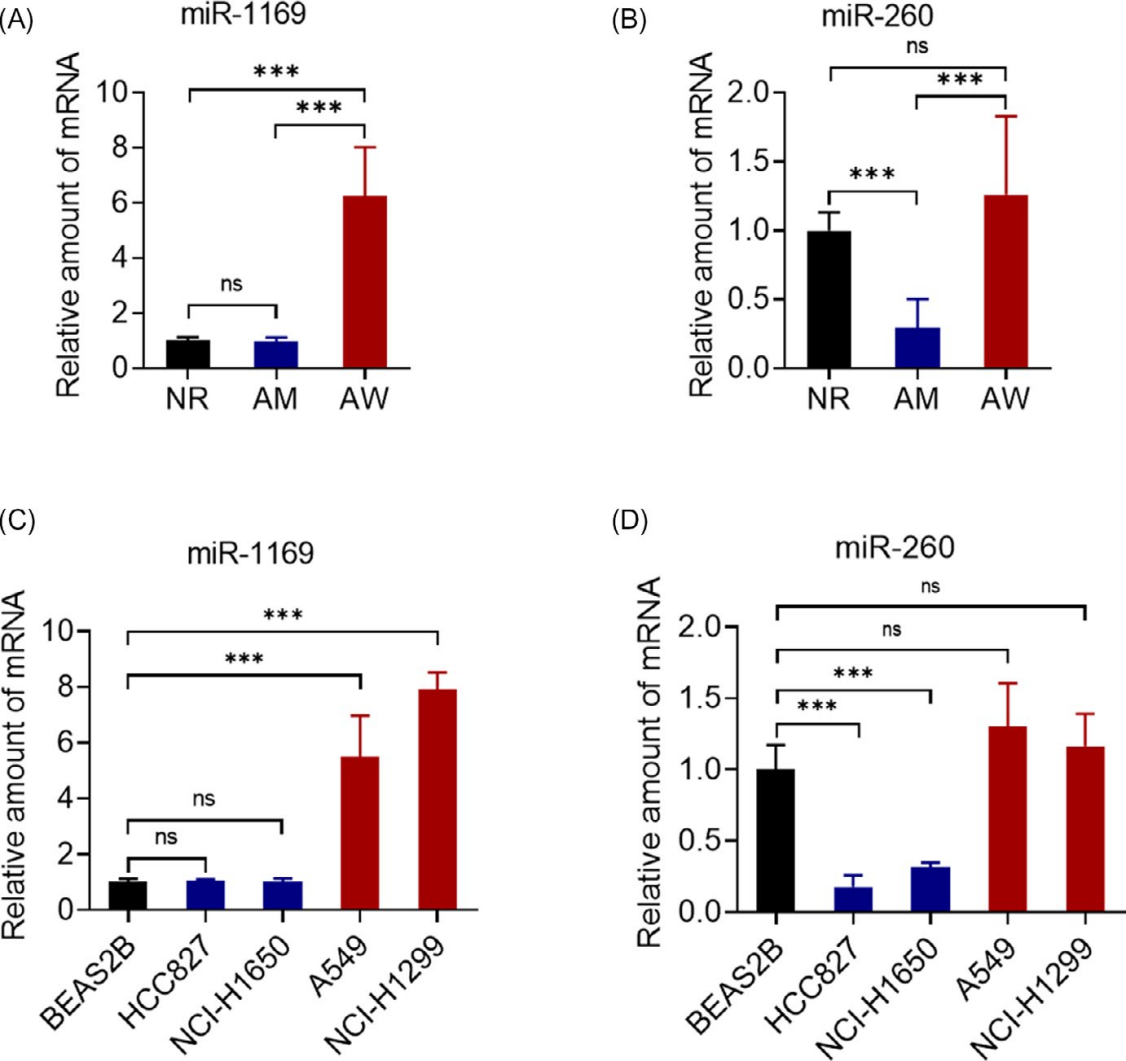
qRT‐PCR data on the expression levels of select miRNAs in the validation set. (A–B) The relative expression of miR‐1169 (A) and miR‐260 (B) for healthy controls (NR, gray), EGFR wild‐type NSCLC patients (AW; red), and EGFR mutant NSCLC patients (AM, purple). (C–D) The relative expression of miR‐1169 (C) and miR‐260 (D) for BEAS2B (NR, gray), HCC827 and NCI‐H1650 (AW; red), and A549 and NCI‐H1299 (AM, purple). Data represent the mean ± SEM. Statistical analysis was performed using the Student's *t* test, ***p < 0.001

To exclude the influence of surgery on the levels of exosomal miRNAs, late‐stage (II‐IV) NSCLC patients with low chances of obtaining suitable surgical procedures were recruited. The NSCLC patients were classified into wild‐type EGFR samples and mutant EGFR samples. The clinical characteristics of 64 selected patients (AW:32; AM:32) are shown in Table 2, and there are no statistical differences in age, gender, and others listed in Table 2. Exosomal miRNAs were obtained using a combination of the ExoQuick kit and the mirVana microRNA isolation system. qRT‐PCR analysis revealed miR‐1169 and miR‐260 expressions in serum exosomes, which were normalized to the miR‐16 internal control gene. Expression of serum exosomal of miR‐1169 in AW NSCLC patients significantly increased 5.8‐fold compared with expression in the control group (Figure 3A). Expression of miR‐260 decreased by 4.3‐fold in serum exosomes of AM NSCLC patients compared with the control group (Figure 3B).

**TABLE 2:** Characteristics of the control, NSCLC groups

Characteristic	Control	NSCLC	
		EGFR mutant	EGFR wild type
Total no	20	32	32
Sex, *n* (%)			
Men	12 (60)	18 (56.3)	22 (68.8)
Women	8 (40)	14 (43.7)	10 (31.2)
Age (mean ± SD)	58.0 ± 13.24	68.2 ± 7.65	65.8 ± 10.1
Stage			
II/III/IV		4/11/17	7/15/10

To investigate the expression of miR‐1169 and miR‐260, we used EGFR mutant cell lines (HCC827 and NCI‐H1650) and wild‐type (A549 and NCI‐H1299) non–small‐cell lung cancer cell lines. Lung epithelial cells, BEAS2B, were used as normal control cells. The Del746‐750 mutation was found in exon 19 of NCI‐H1650 cells, and the L858R mutation was found in exon 21 of HCC827 cells. Both cells lines are sensitive to gefitinib and erlotinib. As shown in Figure 3C, the expression of exosomal miR‐1169 was significantly increased in the culture medium of A549 and NCI‐H1299 cell lines. However, no significant changes in expression were observed in the medium of NCI‐1650 and HCC827 cells. The expression of exosomal miR‐260 was obviously downregulated in the medium of lung cancer cell lines with mutant EGFR, with no significantly changes occurring in the EGFR wild‐type cell lines (Figure 3D). These results demonstrate that the expressions of these miRNAs in the corresponding cell line culture medium are similar to the expression in serum of NSCLC patients.

### 3.4 Exosomal miRNA panels as diagnostic biomarkers in symptomatic patients

**FIGURE 4:**
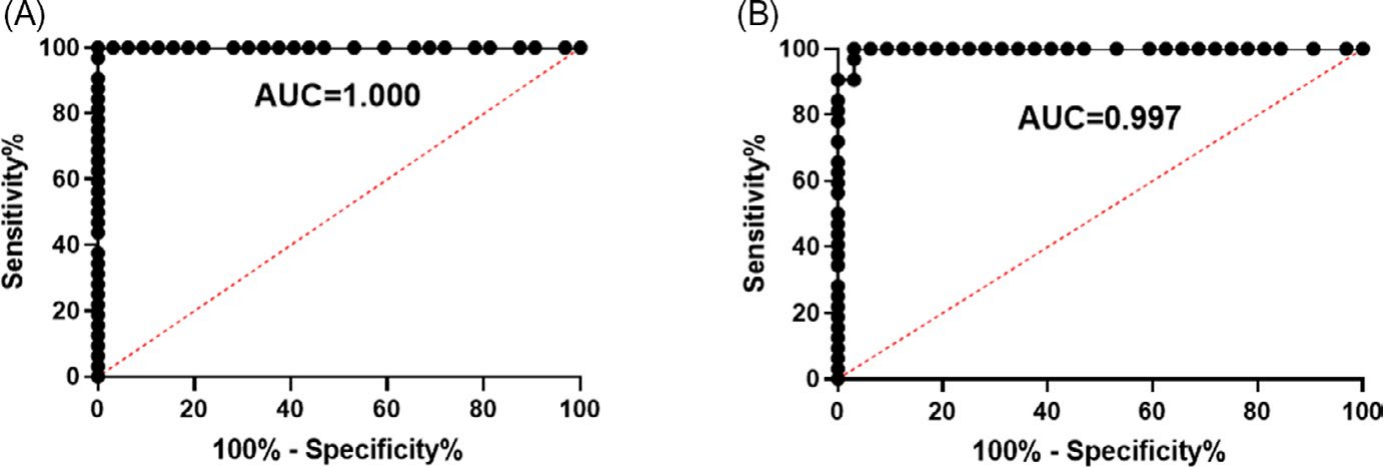
Diagnostic values of exosome miRNAs by qRT‐PCR. The ROC and AUC were used to determine the sensitivity and specificity of selected miRNAs. AUC for miRNAs demonstrates accuracy in differentiating NSCLC patients between AM and AW compared with the symptomatic set of subjects or suspected NSCLC patients in terms of sensitivity and specificity, respectively

To evaluate the early diagnostic value of the miRNA‐sequence profiling data for NSCLC, we selected miR‐1169 and miR‐260 and measured the normalized Ct values using qRT‐PCR in a symptomatic set of 64 subjects (Table 2). The miR‐1169 of NSCLC patients with wild‐type EGFR exhibited an AUC value of 1.000. Wild‐type patients were distinguished from mutant EGFR NSCLC patients by a sensitivity of 80.65% and a specificity of 91.67% (Figure 4A). The miR‐260 of mutant EGFR by NSCLC diagnosis exhibited an AUC value of 0.997 and distinguished mutant EGFR of NSCLC patients from wild‐type EGFR patients by a sensitivity of 83.33% and a specificity of 90.32% (Figure 4B). These findings suggest that these two miRNA panels could serve as primary diagnostic biomarkers for NSCLC diagnosis. By involving NSCLC patients with wild‐type or mutant‐specific miRNAs in the differential diagnosis, further pathologic diagnosis could be improved.

### 3.5 Bioinformatics analysis on selected miRNAs and their targets

**FIGURE 5:**
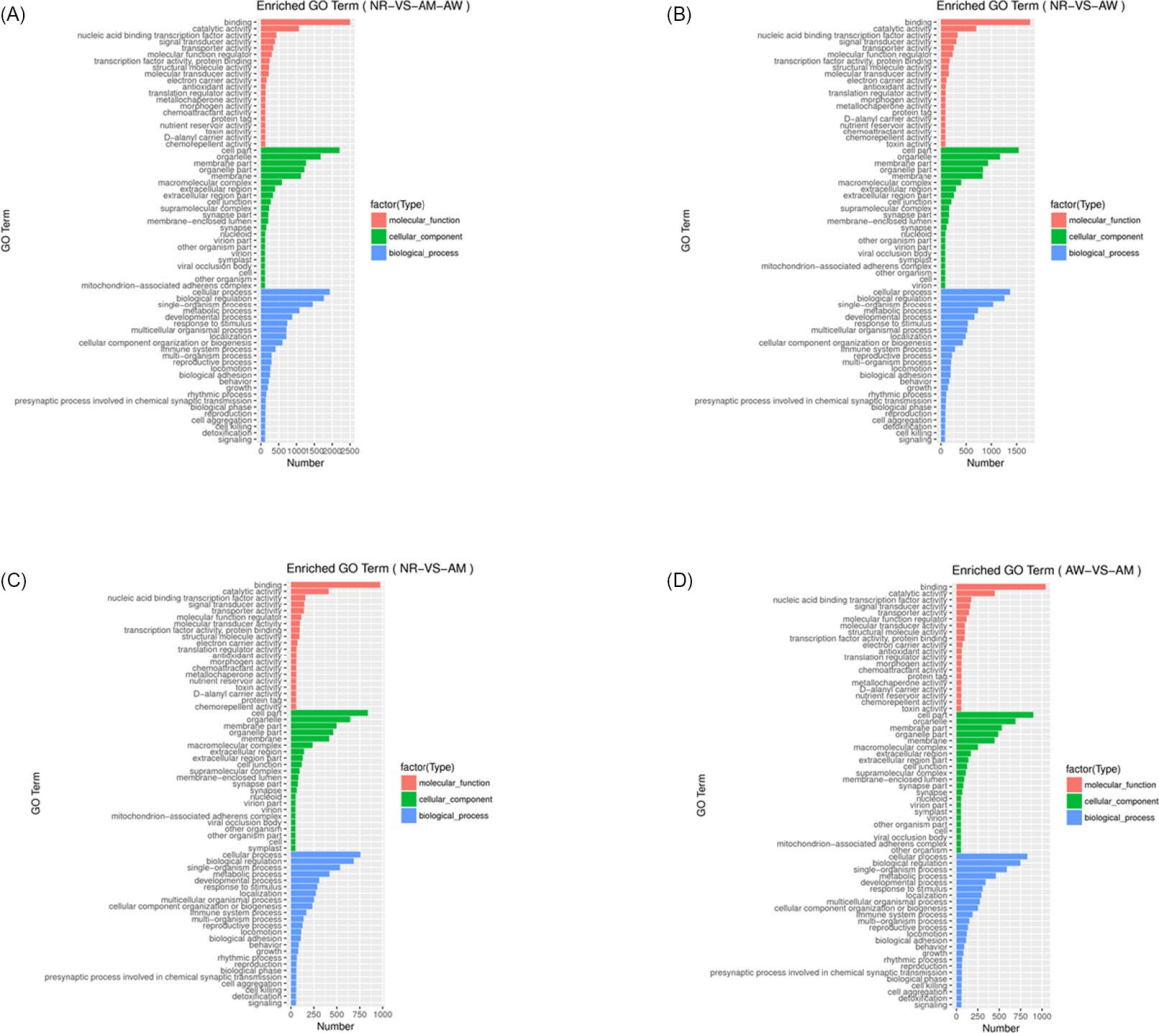
GO pathway analysis and validation of downstream target genes. GO analysis of biological processes, cellular component, and molecule function

**FIGURE 6:**
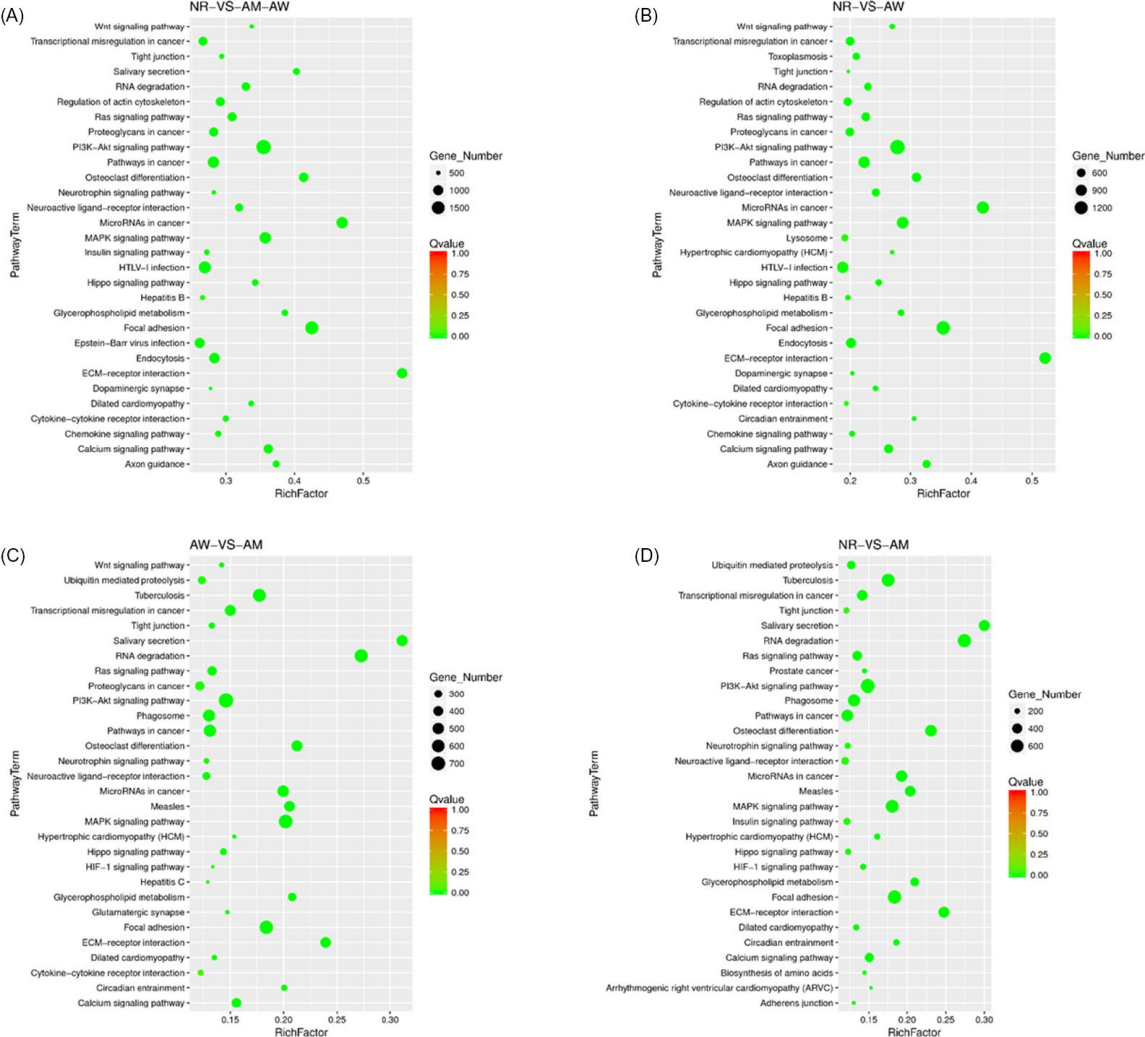
KEGG pathway analysis and validation of downstream target genes. The top thirty KEGG pathways were enriched in the downstream miRNAs target genes

To explore the potential biological roles of these exosomal miRNAs in NSCLC, gene ontology analysis was performed on the molecular function (MFs), cellular component (CCs), and biological processes (BPs). As shown in Figure 5A–D, the top three MFs involved cell adhesion molecule binding, catalytic activity, and nucleic acid binding transcription factor activity. With regard to cellular components, the genes highlighted cell parts, organelles, membranes, and extracellular regions (*p* < 0.001), suggesting that the miRNAs are involved in communication or phagocytosis and exocytosis (Figure 5A–D). The candidate downstream target genes of exosomal miRNAs were mainly enriched for biological regulation, as well as cellular, single‐organism, and developmental processes (*p* < 0.001) (Figure 5A–D). Furthermore, compared with healthy donors, 30 signaling pathways downstream of the miRNA target genes were enriched (*p* < 0.05) in NSCLC patients (Figure 6A). The top six pathways identified were ECM‐receptor interaction, cancer miRNAs, focal adhesion, osteoclast differentiation, salivary secretion, and glycerophospholipid metabolism (Figure 6A). For the EGFR wild‐type samples, the top six pathways selected for analysis include ECM‐receptor interaction, cancer miRNA in cancer, focal adhesion, PI3 K‐Akt signaling pathway, MAPK signaling pathway, and Ras signaling pathway (Figure 6B). The top eight pathways selected for the EGFR mutation samples include salivary secretion, RAN degradation, ECM‐receptor interaction, cancer miRNAs, measles, glycerophospholipid metabolism, MAPK pathway, and focal adhesion (Figure 6C). Finally, significant differences were observed in three pathways between the EGFR mutation and wild‐type samples, namely salivary secretion, RAN degradation, ECM‐receptor interaction (Figure 6D).

## 4 DISCUSSION

Recently studies have shown that exosomes contain small RNAs, in particular miRNAs.^28^, ^29^ Exosomal miRNAs with various biological functions are secreted by primordial cells to silence target mRNAs in other target cells. Exosome miRNAs are involved in the regulation of intracellular signal transduction and gene expression through intercellular communication.^30^ Several detection and purification methods of exosomes or circulating miRNAs have been reported.^31^, ^32^, ^33^ In this study, we extracted the exosomes from plasma of NSCLC patients using exosomal separation systems and observed the characteristic exosome size and shape by TEM and DLS (Figure 1A and B). Expressions of the exosome‐specific CD63 protein was confirmed by western blot analysis (Figure 1C). The high quality of the purified exosomal miRNAs was confirmed using a Bioanalyzer (Figure 1D). The molecular components of exosomes can affect cancer progression by modulating carcinogenesis, growth, invasion, and metastasis. Therefore, the circulating exosomal miRNAs may serve as potential biomarkers for differential disease diagnosis, prognosis, and treatment management.

Exosomal RNA profiling analysis is only feasible after the extraction of high quality RNA. Compared with cellular RNAs, exosomal RNAs are more stable, and are reportedly resistant to physical degradation such as prolonged storage and repeated freeze/thaw cycles. In this study, a comprehensive analysis of NSCLC patients derived exosomal miRNA profiles was performed using next‐generation sequencing. There were 11 and 6 miRNAs expressed at remarkably higher levels, 13 and 8 miRNAs expressed at lower levels in EGFR mutation and wild‐type NSCLC patients, respectively, compared with healthy volunteers (Figure 2, Table 2). Furthermore, 6 EGFR mutation‐dependent and 2 EGFR wild type‐specific tumor‐derived exosomal miRNAs that were expressed at higher levels, and 7 EGFR mutation‐dependent and 2 EGFR wild type‐specific miRNAs that were expressed at significantly lower levels were validated. The reliability of our miRNA‐sequencing data was verified with several proven diagnostic miRNAs for NSCLC and other types of carcinomas, such as let‐7, miR‐21, miR‐24, and miR‐486.^26^, ^34^, ^35^ Novel EGFR mutation‐ and EGFR wild type‐specific biomarkers, such as miR‐361b‐5p and miR‐10b‐5p, were also identified suggesting that they can potentially act as effective indicators in EGFR mutation and EGFR wild‐type diagnosis.

Thus, the serum exosomal level of miRNAs may act as biomarkers of NSCLC, but may not distinguish between AW and AM. The expression of miR‐106b‐3p, miR‐142‐5p, miR‐940, miR‐548ad‐5p, miR‐422, and miR‐1273 has been previously reported as a diagnostic biomarker in other forms of cancer.^36^ However, NSCLC patients with wild‐type EGFR‐specific miR‐1169, and NSCLC patients with mutant EGFR‐specific miR‐260 have not been reported in NSCLC samples.^10^, ^22^, ^37^, ^38^, ^39^ The findings of this study indicated that miR‐1169 and miR‐260 exosomal miRNAs may be specific characteristics to distinguish NSCLC patients with wild‐type EGFR and mutant EGFR in the early cancer stages. Total miRNAs from circulating plasma may be composed of endogenous cellular miRNAs derived from the debris of all kinds of cells. In addition, circulating plasma miRNAs, which are not associated with exosomes, were reported to have differential stability when treated by RNaseA. Those findings suggest that exosomal miRNAs are more suitable for developing diagnostic biomarkers due to their stability and miRNA enrichment. The NSCLC patients with wild‐type EGFR‐ or mutant EGFR‐specific miRNAs validated in this study demonstrate that exosomes are actively excreted from cancer cells and that their specific miRNA components depend on the cells from which they originated.

## CONFLICT OF INTEREST

The authors declare that no conflicts of interests exist.

## Supporting information

Fig S1

## Data Availability

The all of data in this manuscript are availability.
