# Supplementary figures and images for: Serum exosomal microRNAs as predictive markers for EGFR mutations in non–small‐cell lung cancer
Source: J Clin Lab Anal. 2021 Mar 8;35(5):e23743. doi: 10.1002/jcla.23743 (PMC8128312; doi:10.1002/jcla.23743)

Supplementary Figure 1


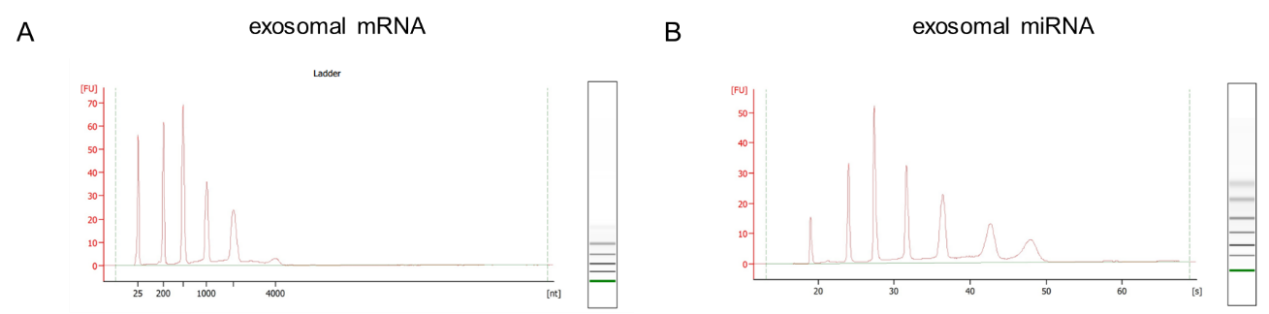

Supplement: Supplementary file 1 — Fig S1 [file JCLA-35-e23743-s001.docx]
